# Supplementary material for: Proteome analyses reveal endoplasmic reticulum stress-induced changes in protein abundance associated with Ube2j2 deficiency in human cell culture
Source: G3 (Bethesda). 2026 Jul 21;16(9):jkag172. doi: 10.1093/g3journal/jkag172 (PMC13535379; doi:10.1093/g3journal/jkag172)

a

GAGCTGAGTTGGAAGGTGCGAGTAGCT//**TCGCATTAAGAAAGACCCGG**TGCCTTACATCTGTGCCGA WT

GAGCTGAGTTGGAAGGTGCGA/ -432 bp-Deletion /**AAGAAAGACCCGG**TGCCTTACATCTGTGCCGA Ube2j2 KO

73 117

b

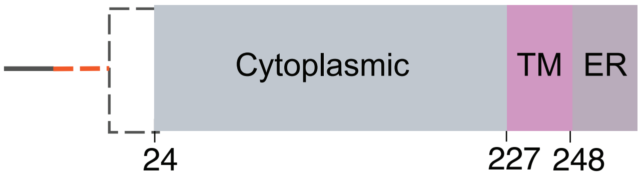

c

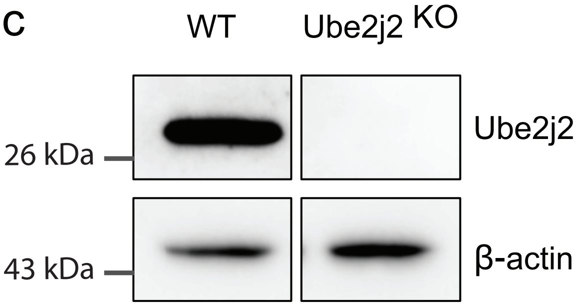

Supplement: Supplementary file 1 [file jkag172_supplementary_data.zip › Supplementary_Figure_1_G3-2026-406927.pdf]
