# Supplementary material for: Proteome analyses reveal endoplasmic reticulum stress-induced changes in protein abundance associated with Ube2j2 deficiency in human cell culture
Source: G3 (Bethesda). 2026 Jul 21;16(9):jkag172. doi: 10.1093/g3journal/jkag172 (PMC13535379; doi:10.1093/g3journal/jkag172)
Supplement: Supplementary file 1 [file jkag172_supplementary_data.zip › Supplementary_Figure_2_G3-2026-406927.pdf]

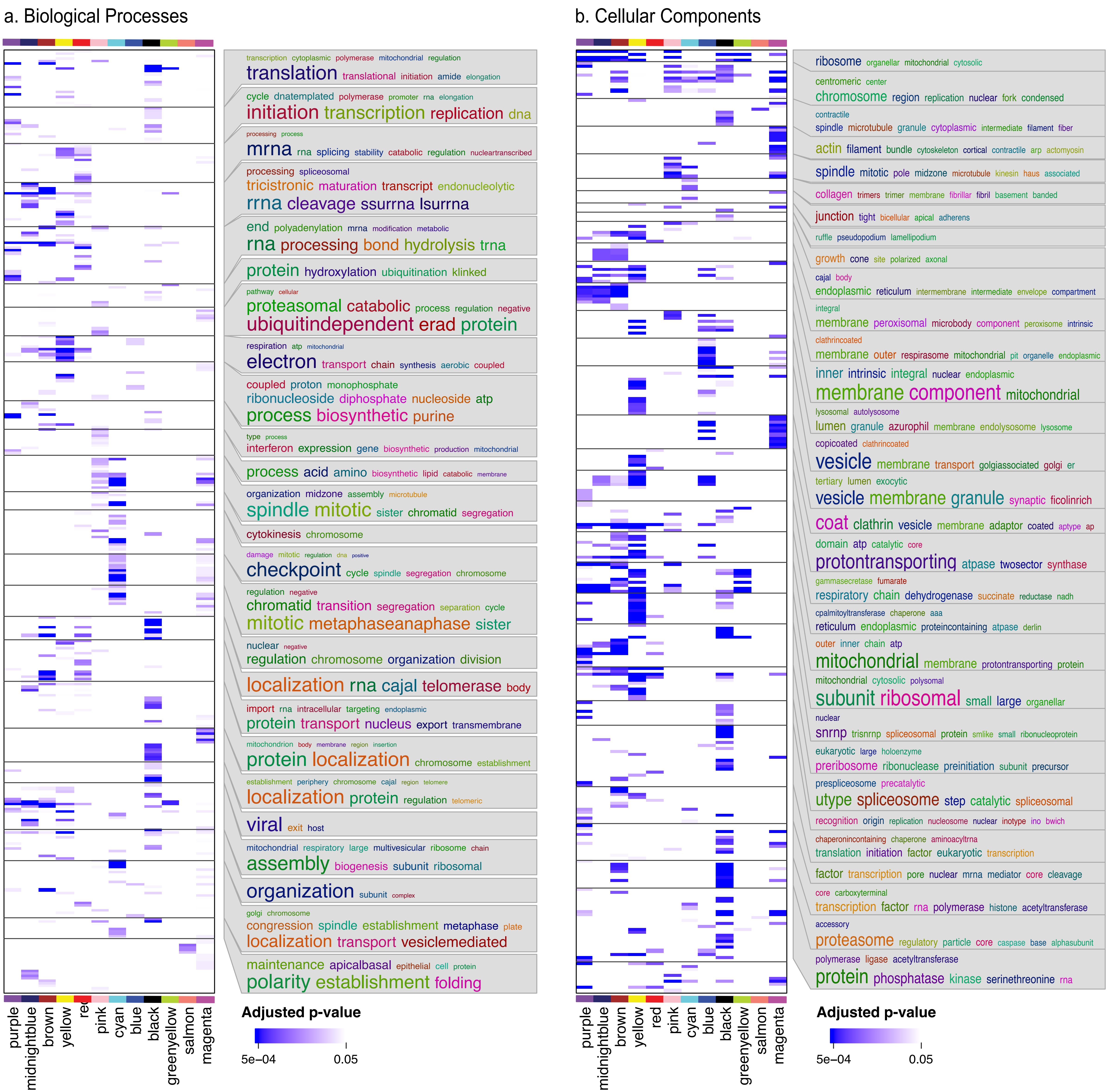

a. Biological Processes
b. Cellular Components
purple
midnightblue
brown
yellow
red
pink
cyan
blue
black
greenyellow
salmon
magenta
Adjusted p-value
5e−04
0.05
translation translational initiation amide elongation
initiation transcription replication dna
mrna rna splicing stability catabolic regulation nucleartranscribed
rrna cleavage ssurrna lsurrna
rna processing bond hydrolysis trna
protein hydroxylation ubiquitination klinked
proteasomal catabolic process regulation negative
ubiquitindependent erad protein
electron transport chain synthesis aerobic coupled
process biosynthetic purine
process acid amino biosynthetic lipid catabolic membrane
spindle mitotic sister chromatid segregation
cytokinesis chromosome
checkpoint cycle spindle segregation chromosome
mitotic metaphaseanaphase sister
regulation chromosome organization division
localization rna cajal telomerase body
protein transport nucleus export transmembrane
protein localization chromosome establishment
localization protein regulation telomeric
viral exit host
assembly biogenesis subunit ribosomal
organization subunit complex
localization transport vesiclemediated
polarity establishment folding
ribosome organellar mitochondrial cytosolic
chromosome region replication nuclear fork condensed
spindle microtubule granule cytoplasmic intermediate filament fiber
actin filament bundle cytoskeleton cortical contractile arp actomyosin
spindle mitotic pole midzone microtubule kinesin haus associated
collagen trimers trimer membrane fibrillar fibril basement banded
junction tight bicellular apical adherens
ruffle pseudopodium lamellipodium
growth cone site polarized axonal
endoplasmic reticulum intermembrane intermediate envelope compartment
membrane peroxisomal microbody component peroxisome intrinsic
membrane outer respirasome mitochondrial pit organelle endoplasmic
inner intrinsic integral nuclear endoplasmic
membrane component mitochondrial
lumen granule azurophil membrane endolysosome lysosome
vesicle membrane transport golgiassociated golgi er
vesicle membrane granule synaptic ficolinrich
coat clathrin vesicle membrane adaptor coated aptype ap
protontransporting atpase twosector synthase
respiratory chain dehydrogenase succinate reductase nadh
reticulum endoplasmic proteincontaining atpase derlin
mitochondrial membrane protontransporting protein
subunit ribosomal small large organellar
snrnp trisnrnp spliceosomal protein smlike small ribonucleoprotein
preribosome ribonuclease preinitiation subunit precursor
utype spliceosome step catalytic spliceosomal
translation initiation factor eukaryotic transcription
factor transcription pore nuclear mrna mediator core cleavage
transcription factor rna polymerase histone acetyltransferase
proteasome regulatory particle core caspase base alphasubunit
protein phosphatase kinase serinethreonine rna
