# Supplementary material for: Proteome analyses reveal endoplasmic reticulum stress-induced changes in protein abundance associated with Ube2j2 deficiency in human cell culture
Source: G3 (Bethesda). 2026 Jul 21;16(9):jkag172. doi: 10.1093/g3journal/jkag172 (PMC13535379; doi:10.1093/g3journal/jkag172)
Supplement: Supplementary file 1 [file jkag172_supplementary_data.zip › Supplementary_Material_Legends_G3-2026-406927.docx]

**Supplementary Material Legends**

**Supplementary Figure 1.** Generation and verification of Ube2j2 KO cell line

(a) Nucleotide sequence of the mutated UBE2J2 clone, illustrating a 432 bp deletion that spans both non-coding (grey) and coding (black) regions. Base numbering is relative to the start of exon 2, the first coding exon. (b) Schematic representation of the Ube2j2 protein domain architecture. The deleted region is indicated by black dashed lines in the protein and by an orange dashed line in the upstream nucleic acid sequence. Numbers refer to amino acid residues; TM: transmembrane domain; ER: endoplasmic reticulum lumen localization. (c) Immunoblot analysis of protein lysates from wild-type (WT) and UBE2J2 knockout (KO) U2OS cells. β-actin serves as a loading control.

ALT TEXT: A figure with three panels labeled a, b and c. a and c show the DNA sequence and schematic representations of the Ube2j2 knock-out in the cells used in this study. c shows the immunoblot that confirmed the successful removal of the Ube2j2 protein in the cells used in this study.

**Supplementary Figure 2.** Overview of significantly enriched GO terms for all modules

(a) Distribution of GO annotations for Biological Processes across all modules. Annotation terms are shown at right, and the statistical significance (adjusted p-value) associated with their assignment to individual proteins is illustrated by the blue bars. (b) Distribution of GO annotations for Cellular Components across all modules, as in (a).

ALT TEXT: A figure with two panels labeled a and b. a and b show significantly enriched GO annotations for proteins in eigen gene modules. The left column of each panel shows the modules (denoted by colors) and the p-values associated with GO assignments to individual proteins. The right column of each panel lists the relevant GO terms that are associated with proteins in at least one module.

**Supplementary File 1.** Module membership

**Supplementary File 2.** Factors defining modules

**Supplementary File 3.** GO annotations in modules

**Supplementary File 4.** Reagent table
